# Relationship between spiritual intelligence and lifestyle with life satisfaction among students of medical sciences

**DOI:** 10.1186/s12909-023-04506-8

**Published:** 2023-07-19

**Authors:** Mojtaba Senmar, Jalil Azimian, Sajad Noorian, Mohaddese Aliakbari, Najmeh Chegini

**Affiliations:** 1 Student Research Committee, Social Determinants of Health Research Center, Research Institute for Prevention of Non–Communicable Diseases, Qazvin University of Medical Sciences, Qazvin, Iran; 2 Department of Statistics, Faculty of Science, University of Qom, Qom, Iran; 3 Emergency Medical Service Center, Qazvin University of Medical Sciences, Qazvin, Iran

**Keywords:** Lifestyle, Spiritual intelligence, Life satisfaction, Students

## Abstract

**Background:**

Young people are the main resources of any country and entering university is considered a sensitive period in their lives. Satisfaction with life is one of the indicators of successful adaptation to life. Lifestyle and spiritual intelligence can complement and give meaning to this variable. The present study was conducted to investigate the relationship between spiritual intelligence and lifestyle with life satisfaction in students of Medical Sciences.

**Methods:**

This descriptive-analytical study was conducted among the students of Qazvin University of Medical Sciences in 2017. One hundred thirty-nine students were included in this study. Data collection tools were the King's spiritual intelligence scale, Miller-Smith lifestyle assessment inventory, satisfaction with life scale (Diener), and demographic information checklist. To analyze the data, descriptive and analytical tests such as independent T-test and one-way analysis of variance and SPSS version 22 software were used. The significance level of all tests was considered less than or equal to 0.05.

**Findings:**

In the present study, 108 were women and the rest were men. 49.6% of students were under twenty years old. The results of this research showed that there is a significant negative relationship between lifestyle and life satisfaction (*r* = -.33) and a significant positive relationship between spiritual intelligence and life satisfaction (*r* = .27) (*p* < 0.05). The mean scores of students in life satisfaction were 22.49 ± 5.92, lifestyle was 49.15 ± 8.35, and spiritual intelligence was 51.59 ± 11.43.

**Conclusions:**

The findings of the present study showed the important role of spiritual intelligence and lifestyle in students' life satisfaction. These findings can be used by administrators and policymakers in formulating interventions and providing a suitable context to improve students' life satisfaction.

## Introduction

Students constitute a large part of the young and productive population of any country, who are usually exposed to unhealthy physical and mental behaviors more than other people due to their special conditions [1]. Since they are growing up in a new social environment and experiencing a certain level of independence from their parents [2]. There are different challenges in medical sciences, such as job burnout, high stress, and dissatisfaction in the profession, which also include other aspects of the lives of students in these fields [3]. These situations, along with challenges such as fear of the future and balancing academic and social life, affect the satisfaction of these students' lives [4].

Life satisfaction is a measure of mental well-being that evaluates a person's feelings and attitudes about life at a certain point in time [5]. This concept is one of the most important indicators of successful adaptation to life and can be useful for health, longevity, and social relationships [6]. Income, job satisfaction, resilience, and support have been identified as positive predictors of life satisfaction, while unemployment, stress, anxiety, and depression have been shown that reduce life satisfaction [6]. Adaptation to faculty, interpersonal conflicts between students and teachers, exposure to death and suffering, personal life events, and curricular factors (eg, new ways of learning) are some of the things that medical science students experience [3]. And this is how satisfaction with life as one of the strong indicators of quality of life [7] is under the influence of various factors [4]. It seems that one of the important things in this regard is the revival or modification of these people's lifestyle [4].

Lifestyle is one of the concepts that is widely used in various social, economic, and cultural fields [8]. Lifestyle is the pattern of actions by which people are distinguished from each other, and these patterns gradually become the normal routine of daily life [8]. The student period is considered a bridge between adolescence and adulthood, and the stabilization of behaviors, habits, and lifestyle in general during this period will affect the whole life of a person [4]. Behaviors and lifestyle habits learned in childhood appear in early adolescence or youth and will be established in adulthood [2]. Although correcting improper habits will be difficult and time-consuming, with early detection of these habits in early adolescence and fact during student life and attention and follow-up, it is possible to change or correct them in time [2]. Besides, it is important to note that one of the cases that enable people to solve problems and achieve goals is spiritual intelligence [9].

Spiritual intelligence refers to a set of spiritual capacities and resources, the use of which in daily life increases a person's adaptability and satisfaction with life [8]. This concept, which affects performance, solving problems, and finding a purpose in life [10], is also effective in increasing mental health and reducing occupational stress [11]. During the years of study, students generally face different academic, social, and personal needs [10]. They are very vulnerable due to their academic and special social status during their student days and changing the social environment, establishing new relationships, and academic stress can change their lifestyle [10]. But spiritual intelligence plays an important role in helping them [11]. This concept includes the highest levels of development in different emotional, moral, cognitive, and interpersonal fields and helps a person to adapt to the phenomena around him [11].

Spirituality is a useful strategy in situations beyond the individual's control [12]. The results of Salmani et al.'s study among students of medical sciences showed that there is a direct relationship between spiritual attitude and life satisfaction [12]. The results of Wachholtz and Rogoff's study among medical students also showed that students who have higher spiritual health are more satisfied with their lives [13], but there are also contradictory results at the University of Birmingham that do not show a relationship between spirituality and life satisfaction [14]. Francis and Hales also reported little or no relationship between prayer frequency and life satisfaction among undergraduate students [15].

Few studies have been conducted on the relationship between lifestyle and life satisfaction among students of medical sciences. However, Machol et al.'s study among students of various medical sciences conducted in Poland showed that lifestyle has a positive relationship with life satisfaction [16]. Badura-Brzoza et al.'s study among medical students also showed that there is a positive relationship between life satisfaction and health-promoting behaviors [17]. The results of Bosing et al.'s study in one of the American private universities showed that there is no significant correlation between any of the the health-related components of fitness and life satisfaction scores [18]. However, excessive use of the internet is associated with various psychological problems such as depression, self-harm, sleep deprivation, lack of exercise, and loss of concentration [19]. But Alsaad and AlMukhtar's study among medical students showed that contrary to the findings of some countries, medical students in Saudi Arabia have a better quality of life with moderate levels of internet addiction [19].

Therefore, paying attention to the student group as a unique and constructive human force of a country, who are in the era of ideals, grandiose thoughts, and hopes, is considered a strategy to maintain mental and physical health for other members of the society as well [20, 21]. This issue is more important for students of medical sciences because they will directly affect people's health in the near future. Therefore, the present study was conducted to determine the relationship between spiritual intelligence and lifestyle with life satisfaction in students of Qazvin University of Medical Sciences (QUMS).

## Methods

### Study design, participants and sample size

This descriptive-analytical study was conducted among the students of QUMS in 2017. Considering the confidence level of 95% and the test power of 80%, 139 students were included in the study. According to the statistics of the education department, the total number of university students is 2602. Using the quota sampling method, 14 students from the Faculty of Medicine, 35 students from the Faculty of Paramedicine, 43 students from the Faculty of Health, 21 students from the Faculty of Nursing, and 26 students from the Faculty of Dentistry were included in the study. The inclusion criteria included the willingness to participate in the research, and the samples were not included in the study if they had a confirmed psychological illness. Incomplete completion of the questionnaires was the exclusion criterion of the study.

### Data collection method

The participants were selected from among the students who have the inclusion criteria. Explanations about the purpose and method of doing the work were presented to them, and signed an informed consent form before data collection. Questionnaires were given to them by the researcher. While emphasizing the confidentiality of all information, they were requested to answer all questions completely. Students completed 4 tools. All the tools were written and completed in a calm and quiet environment. Participants were allowed to refuse to answer some items or to withdraw from the study without consequences. The duration of completing the tools was between 60–90 min.

### Data collection tools

#### Section 1

A checklist was used to collect demographic information. This checklist included: age, sex, marital status, college, religion, native status, father's education level, mother's education level, current residence, relationship with religion, and family income level relative to expenses.

#### Section 2

King's spiritual intelligence scale was used to assess spiritual intelligence. The scale was developed by David King based on the model of spiritual intelligence [22, 23]. This scale has 24 items that measure spiritual intelligence based on a 5-point Likert scale: "Completely disagree with a score of 0, disagree with a score of 1, have no opinion with a score of 2, agree with a score of 3, and completely agree with a score of 4" (0–4). Its scores range from zero to 96. The higher the score in this scale, the higher the spiritual intelligence [24]. Its reliability was reported in King's study on 619 students based on the alpha coefficient of 0.95 [25]. In Iran, its reliability was estimated using Cronbach's alpha coefficient of 0.88. Face and content validity were confirmed by psychological experts. Exploratory factor analysis and confirmatory factor analysis were used to calculate the construct validity of the scale. The obtained results showed that this scale is a reliable scale for measuring spiritual intelligence, and due to the appropriate reliability, it can be used in educational and research environments [26]. The internal reliability of the scale in the present study with Cronbach's alpha was 0.88.

#### Section 3

In the present study, lifestyle data were obtained using the Miller-Smith lifestyle assessment inventory [27]. Miller and Smith developed this 20-question inventory and confirmed its validity and reliability (Cronbach's alpha: 0.86) [27]. Each question has five answers (1 = always, 2 = often, 3 = sometimes, 4 = rarely, 5 = never). The range of scores is between 20–100, and a higher score indicates an unpleasant and unhealthy lifestyle. In this inventory, a score of less than 50 is considered an excellent/healthy lifestyle, 50–70 is moderate/very good, 70–95 is mild/good and more than 95 is considered unhealthy/poor [28]. The validity of the inventory was confirmed by the members of the medical faculty of Isfahan (Iran) and its reliability in a pilot study on twenty pulmonary patients was 0.86 and Cronbach's alpha of each question was higher than 0.05 [29]. In the above research, to obtain the reliability of the lifestyle assessment inventory, it was administered to 30 people at an interval of two weeks, and its reliability was obtained using Cronbach's alpha coefficient of 0.85 [29]. In the current study, the internal reliability of the inventory was good (Cronbach's alpha = 0.84).

#### Section 4

In this section, the life satisfaction scale (SWLS) was used, which is theoretically designed to measure a person's overall judgment of life satisfaction [30]. This scale was developed by Diener et al. [31]. The scale has a total of 5 items designed in the form of 7-point Likert scale (1 = strongly disagree, 2 = disagree, 3 = slightly disagree, 4 = neither agree nor disagree, 5 = slightly agree, 6 = agree, 7 = strongly agree). The total scores are determined in the range of 5–35 and higher scores indicate higher satisfaction [30, 31]. Scores as very satisfied [31–35], satisfied [26–30], slightly satisfied [21–25], neutral [20], slightly dissatisfied [15–19], dissatisfied [10–14], and very dissatisfied [5–9] are classified [32]. The reliability of the scale using Cronbach's alpha method is 0.83 and 0.69 with the test–retest method [33]. The construct validity of the scale has been evaluated through convergent validity using the Oxford Happiness Inventory and the Beck Depression Inventory, and this scale has shown a positive correlation with the Oxford Happiness Inventory and a negative correlation with the Beck Depression Inventory [33]. In the present research, based on the calculation of the internal consistency of the scale, the alpha was 0.86.

### Data analysis

The analysis was done by using SPSS software, version 22, and with the help of descriptive and inferential tests. To examine the variables of study in two stated variables, the independent T-test was used, and for more than two stated variables, the one-way comparative analysis. Pearson’s correlation test was conducted to examine the relationship between variables. The significance level for all tests was considered to be 0.05 or lower.

### Findings

A total of 139 students of QUMS completed the questionnaires. 22.30% of the samples were men and 77.70% were women. About half number of the studied samples were under 20 years old (49.6%). Other demographic information is given in Table 1. The mean scores of students in life satisfaction were 22.49 ± 5.92, lifestyle was 49.15 ± 8.35, and spiritual intelligence was 51.59 ± 11.43. The results of the correlation test showed that there is an inverse and significant statistical correlation between lifestyle and life satisfaction (*p*-value = 0.000)—(*r* = -0.33). A simple linear regression model was used to model satisfaction with life (Y) based on lifestyle (X). The regression model between these two variables is estimated using the table of regression coefficients as follows: Y = 34.220–0.239 X.

**Table 1:** Demographic information of the participants (*N* = 139)

Characteristic	Category	%	N
Age	< 25	49.64	69
	21–25	43.88	61
	> 25	6.47	9
Sex	Women	77.70	108
	Men	22.30	31
Marital Status	Single	89.20	124
	Married	10.80	15
Grade	Associate Degree	4.31	6
	Bachelor	64.02	89
	Master	2.87	4
	Doctoral	28.77	40
College	Dental	18.70	26
	Medical	10.07	14
	Nursing	15.10	21
	Paramedical	25.17	35
	Health	30.93	43
Religion	Shia	97.841	136
	Sunni	2.158	3
Native Status	Yes	45.32	63
	No	54.67	76
Father's Education Level	High School	25.89	36
	Diploma	41.00	57
	University	33.09	46
Mother's Education Level	High School	30.93	43
	Diploma	48.92	68
	University	20.14	28
Current Residence	University's Dormitory	50.35	70
	Family Home	41.72	58
	R u Single	7.91	11
Family Income Level Relative to Expenses	Weak	3.59	5
	Medium	52.51	73
	Good	41.72	58
	Excellent	2.15	3

In addition, the results showed that there is a direct and significant relationship between spiritual intelligence and life satisfaction (*p*-value = 0.001, *r* = 0.27). A simple linear regression model was used to model life satisfaction (Y) based on spiritual intelligence (X). The regression model between these two variables is estimated using the table of regression coefficients as follows: Y = 0.178 + 15. 142 X. Pearson's correlation coefficient showed that lifestyle and spiritual intelligence do not have a statistically significant relationship with the age variable, but life satisfaction has a statistically significant relationship with age (*r* = 0.19, *p*-value = 0.02). Tables 2, 3 and 4 show the comparison of the mean scores of three variables based on demographic information.

**Table 2:** Mean and standard deviation of lifestyle, spiritual intelligence and life satisfaction according to demographic information of the participants (*N* = 139)

Variable	Sex				*P*-Value	Mean Equality Test Statistic
	Men		Women			
	Mean	Standard Deviation	Mean	Standard Deviation		
Lifestyle	50.29	8.07	48.82	8.44	.391	-.86
Spiritual Intelligence	51.90	12.11	51.50	11.295	.866	-.168
Life Satisfaction	21.29	6.26	22.84	5.80	.200	1.289
Variable	Marital Status				*p*-value	Mean Equality Test Statistic
	Single		Married			
	Mean	Standard Deviation	Mean	Standard Deviation		
Lifestyle	48.53	9.28	49.22	8.27	.763	.302
Spiritual Intelligence	51.06	11.89	51.66	11.43	.850	.189
Life Satisfaction	24.60	5.08	5.98	22.24	.146	-1.462
Variable	Religion				*P*-Value	Mean Equality Test Statistic
	Sunni		Shia			
	Mean	Standard Deviation	Mean	Standard Deviation		
Lifestyle	50.33	4.50	49.12	8.42	0.805	-0.247
Spiritual Intelligence	53.66	12.22	51.55	11.46	0.753	-0.316
Life Satisfaction	25.33	4.04	22.43	5.95	0.404	-0.837
Variable	Native Status				*P*-Value	Mean Equality Test Statistic
	No		Yes			
	Mean	Standard Deviation	Mean	Standard Deviation		
Lifestyle	49.65	8.06	48.53	8.72	.434	-.784
Spiritual Intelligence	50.61	9.73	52.77	13.19	.269	1.109
Life Satisfaction	23.35	5.57	21.46	6.20	.060	-1.895

**Table 3:** Mean and standard deviation of lifestyle, spiritual intelligence and life satisfaction according to demographic information of the participants (*N* = 139)

Variable	Relationship With Religion			*P*-Value	Test Statistics
	Good	Average	Poor		
Lifestyle	48.01	50.84	47.00	0.135	2.030
Spiritual Intelligence	54.48	48.22	39.66	0.001	7.211
Life Satisfaction	23.6	21.29	16.00	0.012	4.586
Variable	Father's Education Level			*P*-Value	Test Statistics
	University	Diploma	High School		
Lifestyle	46.86	50.1	50.55	.074	2.657
Spiritual Intelligence	54.41	50.19	50.22	.124	2.117
Life Satisfaction	22.71	23.19	21.11	.246	1.419
	Mother's Education Level			*P*-Value	Test Statistics
	University	Diploma	High School		
Lifestyle	49.50	48.44	50.04	.600	.513
Spiritual Intelligence	53.85	50.76	51.44	.485	.728
Life Satisfaction	21.89	22.66	22.62	.835	.180
Variable	Current Residence			*P*-Value	Test Statistics
	R u Single	Family Home	University's Dormitory		
Lifestyle	53.72	49.29	48.31	.134	2.039
Spiritual Intelligence	49.27	53.50	50.38	.243	1.431
Life Satisfaction	24.81	21.79	22.71	.274	1.307

**Table 4:** Mean and standard deviation of lifestyle, spiritual intelligence and life satisfaction according to demographic information of the participants (*N* = 139)

Variable	Family Income Level Relative to Expenses				*P*-Value	Test Statistics
	Excellent	Good	Medium	Weak		
Lifestyle	41.00	48.56	49.79	51.40	.271	1.319
Spiritual Intelligence	56.66	52.44	50.38	56.40	.459	.869
Life Satisfaction	29.66	23.36	21.93	16.40	.008	4.127
Variable	Grade				*P*-Value	Test Statistics
	Doctoral	Master	Bachelor	Associate Degree		
Lifestyle	52.62	41.50	48.47	41.16	.001	2.653
Spiritual Intelligence	54.87	60.00	49.87	49.66	.053	.362
Life Satisfaction	24.25	27.50	21.64	20.16	.026	2.653

## Discussion

The findings of the present study, which investigated the relationship between spiritual intelligence and lifestyle with life satisfaction in students of QUMS showed that there is an inverse and significant statistical correlation between lifestyle and life satisfaction. This means that with the increase in lifestyle scores, the level of satisfaction with life decreases. Considering that the increase in lifestyle scores means an unpleasant lifestyle, it should be said that the more unpleasant lifestyle is, the lower the scores of students' satisfaction with life.

In the study of Phulkerd et al. conducted on people over 60 years old [34], the overall score of life satisfaction of people was 24.2, and life satisfaction has a statistically significant relationship with various lifestyle variables. In other words, the results of the two studies are in line with each other, although the life satisfaction scores of the above study are higher than the mean scores of the present study. The results are also consistent with the study of Machul et al. [35]. In a single study by Vahedi Kojnagh et al. [36], life satisfaction has a statistically significant relationship with a health-promoting lifestyle. In other words, the results of the two studies are in line with each other, although Kojnagh et al. consider the health-promoting lifestyle and the mean life satisfaction are lower (18.77) than the mean scores of the present study. Life satisfaction reflects the balance between a person's wishes and his current situation and is considered a cognitive, well-being, and mental component. Therefore, it can be said that life satisfaction in old age is influenced by Review of past life and the low life satisfaction scores in the elderly in the above study can be explained to some extent. Because students are in young age groups, this component has not yet been exposed to various variables.

In the study of Kim and Yang on Korean adults [37], it has been determined that life satisfaction is a mediator variable of a high healthy lifestyle and perceived physical attractiveness. In other words, it can be said that the results are somewhat consistent with the results of the present study. In the study of Porhadi and Torkashvand [38], who examined the relationship between lifestyle and marital satisfaction, the results showed that these variables have a significant relationship with each other. Although the lifestyle scores in the above study (62.88) were higher than the mean lifestyle scores of the present study. This difference in scores is probably due to the type of study samples that included only married people, while in the researchers' study, most of the samples were single people. In the study of Motevalian et al. [39], it was also found that the mean score of life satisfaction in students is 21.26, which is somewhat close to the results of the present study.

The present study showed that there is a direct and significant relationship between spiritual intelligence and life satisfaction. The results of Baloochi et al.'s study [10] showed that spiritual intelligence has a significant and inverse relationship with anger, and the spiritual intelligence of medical students in this study is 52.28. Although this study did not examine the relationship between spiritual intelligence and life satisfaction, the results of the mean scores of spiritual intelligence are in line with the results of the present study and have little differences from each other. It should be said that spiritual intelligence does not only include capacity but exists as a set of mental capacities distinct from personalities and behavioral experiences that correspond to established standards of intelligence [40]. Therefore, in the study of Liu et al. among primary school teachers [41], it was found that spiritual intelligence and life satisfaction have a positive and significant relationship, which is in line with the results of the present study.

Ansari et al.'s study among university faculty professors [42] showed that there is no significant relationship between spiritual intelligence and job satisfaction. Although this study did not consider life satisfaction, it can be said that job satisfaction is one of the important factors in the discussion of life satisfaction. With these differences, it should be said that the results of the above study are not in line with the researchers' study. In addition, the spiritual intelligence scores of Ansari et al.'s study are also higher than the scores of students in the present study (basic science professors 57.60 and clinical professors 70.54). Perhaps this increase can be attributed to the statistical population that the above study was conducted among professors, and of course, the increase in age, academic degree, and life situations can increase the spiritual intelligence of people.

The results showed that spiritual intelligence in people who have a good relationship with religion is more than in other groups and the differences are significant. Spiritual intelligence is a form of intelligence that combines the concepts of spirituality and intelligence in a new structure and has provided humanity with the rational use of spiritual skills to search for meaningful and valuable issues. This intelligence, which is the basis of human values, beliefs, and actions, enables him to search for growth and excellence in the face of the realities of life by focusing on ontological issues [43]. Therefore, the increase in spiritual intelligence scores of people who have a good relationship with religion can be justified.

In the present study, the mean scores of spiritual intelligence in other demographic variables were not significant and did not differ. Contrary to the present study, in Baloochi et al.'s study [10], it was found that age has a direct and significant relationship with spiritual intelligence, and the difference in spiritual intelligence scores is significant according to gender, marital status, and type of college. It can be said that as a person's age increases throughout his life, his understanding of phenomena and experiences changes, and it can also affect his spiritual intelligence. In the study of Mohammadi et al. [44], it was also found that spiritual intelligence has no significant relationship with marital status, place of residence, and sex, which is in line with the findings of the present study. Contrary to the present study, in Chenarani et al.'s study [45], it was found that religion has a significant relationship with spiritual intelligence. Although, unlike the above study, in the present study, the spiritual intelligence of Sunni students is higher than the spiritual intelligence of other people.

The results of the study showed that the mean scores of lifestyle in different faculties and educational levels have significant differences from each other, and students in the doctoral course and medical school students have the highest scores in this variable. In other words, it should be said that medical and doctoral students have a more unfavorable lifestyle than other groups. In a study, lifestyle shows a significant relationship with the academic field, in other words, the results are in line with the researchers' study [46]. Many students in the university turn to excessive behaviors such as alcohol consumption, smoking, and unhealthy eating habits and adopt unhealthy and inappropriate ways [47]. It seems that this increase in lifestyle scores is related to the volume of courses and the duration of their studies. Because studying for a doctorate and the medical field is more difficult than in other fields.

In line with the results of the present study, in the study of Sadeghi et al. [47], it was also found that lifestyle has no significant relationship with the place of residence. Contrary to the results of the researchers' study, in another study, a significant relationship was observed between health-oriented lifestyle-promoting behaviors and a mother's education and sex [46]. Although the variable of health-promoting behaviors is different from lifestyle, it must be said that these behaviors move in the direction of improving a desirable and suitable life, and therefore it is not in line with the results of the present study. One of the reasons for this difference can be attributed to the study samples because the above study was conducted among Christians and the present study is among Muslim people.

In the study of Zehni and Rokhzadi [48], it was found that lifestyle has no significant relationship with the father's education, place of residence, and marital status, which is in line with the results of the present study. Religion is a system of action based on beliefs that have been sent by God to humans on the path of growth and excellence in the realm and intellectual and social dimensions [49]. In the study of Habibi Kaleybar et al. [49], it was found that there is a negative and significant relationship between internal religious orientation and high-risk behaviors. The internal religious orientation and the relationship with religion variable expressed by the samples of the present study are somewhat similar in terms of meaning, and risky behaviors show an undesirable and unhealthy lifestyle. Therefore, it can be said that the results of the two studies are not consistent. Perhaps one of the reasons for these differences can be attributed to the type of students in the two studies because the present study was conducted on students of medical sciences, while the study by Kaleybar et al. was not conducted on medical students.

The results of the study showed that the mean scores of life satisfaction according to the variable of family income to cost ratio, educational level and relationship with religion have significant differences from each other. Also, the results showed that there is a direct and significant relationship between life satisfaction and age. In line with the present study, in the study of Gavin-Chocano et al. [50], age has a statistically significant relationship with life satisfaction. However, in the above study, people were divided into two age groups, over 25 years old and under 25 years old, and people under 25 years old got high scores.

In a study among Christians, it was found that life satisfaction has a negative relationship with negative feelings toward God. Participants who were more satisfied with life felt more religiously comfortable and considered their relationship with God to be close and cooperative [51]. which is consistent with the results of the present study. In the study of Ziakhodadadian et al. [46], it was found that nurses who reported their economic status as poor reported lower life satisfaction, which is in line with the results of the present study. Unlike the present study, in the study of Rogowska et al. [6], it was found that sex has a significant relationship with life satisfaction in German and Russian students. And Russian men and German women scored higher. However, in the present study, women scored higher than men. In line with the present study, in the study of Rogovaska et al., Czech and Polish master's students have higher life satisfaction scores than undergraduate students.

### Limitations

One of the limitations of the study is the generalization of the results Because the situations of students in diverse universities are probably different from each other. In addition, students of medical sciences may experience different conditions due to the nature of their field.

### Strengths

The variety of study samples is the strength of this study. Because the sample from all faculties of the university has been included in the study.

## Conclusions

The findings of the study among students of medical sciences showed that spiritual intelligence and lifestyle have a significant direct and inverse relationship with life satisfaction, respectively. In addition, spiritual intelligence and satisfaction with life in students are higher than mean, while they are higher than mean in undesirable and inappropriate lifestyles. According to the results, it is suggested that related professionals include solutions in educational and cultural planning to improve lifestyle, improve spiritual intelligence and finally improve the level of satisfaction with life in these students. Related policymakers and managers are expected to provide a suitable platform for improving life satisfaction. It is suggested to conduct a comparative study of variables between medical and non-medical sciences students in future studies. The results of the present study can be used in similar contexts.

## Data Availability

Data are available by contacting the corresponding author.

## Abbreviations

SWLS: Life Satisfaction Scale
QUMS: Qazvin University of Medical Sciences
